# A comprehensive guide to study the agreement and reliability of multi-observer ordinal data

**DOI:** 10.1186/s12874-024-02431-y

**Published:** 2024-12-20

**Authors:** Sophie Vanbelle, Christina Hernandez Engelhart, Ellen Blix

**Affiliations:** 1 Methodology and Statistics, CAPHRI, Maastricht University, P. Debyeplein, 1, Maastricht, 6229 HA The Netherlands; 2 Norwegian Research Center for Women’s Health, Oslo University Hospital, P.O box 4950 Nydalen, Oslo, N-0424 Norway; 3 Faculty of Health Sciences, Oslo Metropolitan University, P.O box 4 St Olavs plass, Oslo, N-0130 Norway

**Keywords:** Clinical test validation, Reproducibility, Repeatability, Guideline, Reliability, Agreement, Measurement error

## Abstract

**Background:**

A recent systematic review revealed issues in regard to performing and reporting agreement and reliability studies for ordinal scales, especially in the presence of more than two observers. This paper therefore aims to provide all necessary information in regard to the choice among the most meaningful and most used measures and the planning of agreement and reliability studies for ordinal outcomes.

**Methods:**

This paper considers the generalisation of the proportion of (dis)agreement, the mean absolute deviation, the mean squared deviation and weighted kappa coefficients to more than two observers in the presence of an ordinal outcome.

**Results:**

After highlighting the difference between the concepts of agreement and reliability, a clear and simple interpretation of the agreement and reliability coefficients is provided. The large sample variance of the various coefficients with the delta method is presented or derived if not available in the literature to construct Wald confidence intervals. Finally, a procedure to determine the minimum number of raters and patients needed to limit the uncertainty associated with the sampling process is provided. All the methods are available in an R package and a Shiny application to circumvent the limitations of current software.

**Conclusions:**

The present paper completes existing guidelines, such as the Guidelines for Reporting Reliability and Agreement Studies (GRRAS), to improve the quality of reliability and agreement studies of clinical tests. Furthermore, we provide open source software to researchers with minimum programming skills.

**Supplementary Information:**

The online version contains supplementary material available at 10.1186/s12874-024-02431-y.

## Background

In many medical fields, such as obstetrics, decisions for clinical interventions are guided by assessments based on health professionals’ observations and examinations. Agreement and reliability are therefore two important concepts. Agreement refers to the closeness of observations made by the same (intra-observer) or by different (inter-observer) observers on the same patients/objects [1], such as midwives or obstetricians. Disagreements can have consequences for the choice of treatment. For example, when assessing fetal well-being during birth by interpreting and classifying an intrapartum cardiotocography (CTG), differences between midwives or obstetricians could lead to different decisions for clinical management (e.g., excessive, inappropriate or lack of appropriate intervention). Reliability refers to the ability of a measurement instrument to differentiate between patients/objects within a particular population despite measurement error. Reliability is a population characteristic, that depends on the heterogeneity of the population [2]. Unreliable measurement instruments lead to inconsistency when a measurement is repeated. Such measurement instruments are therefore useless, as they cannot assess any change in patients (e.g., fetal heart rate change).

During a literature review on the assessment of reliability and agreement in intrapartum CTGs [3], we noted four main problems persisting in the scientific literature when an agreement or a reliability study is performed on ordinal data, namely, 1) the words agreement and reliability were used interchangeably , while they are two distinct concepts for which different statistical measures are defined; 2) the choice of specific statistical measures were seldom justified; 3) values of agreement and reliability were interpreted independently of the statistical measure used and 4) sample size calculations were seldom performed.

The concept of reliability emerged in the early 20th century, with the development of the classical test theory. In classical test theory, an observed score is decomposed in a true score and an error component [4]. In this framework, reliability is defined as the squared correlation between the observed and the true scores. Since the true score is generally unknown, it is often estimated by making replicated measurements on patients or objects (see [5] for a detailed discussion). Lord and Novic [2] demonstrated that, under the specific mathematical assumption of parallelity, reliability in classical test theory corresponds to the correlation between the replicated measurements. The concept of reliability was later extended, primarily using variance partitioning techniques, in various ways by altering the mathematical assumptions and methods for obtaining replicated measurements (see e.g., [6]). Although initially developed for quantitative scales, the same mathematical models are commonly applied to binary and ordinal scales. The concept of agreement is closely related to the notion of distance between replicated measurements. Rather than developing the concept of agreement at the population level, agreement measures were developed on a ad hoc basis as descriptive measures in a sample. Many statistical measures were introduced depending on the measurement level of the scale, and some desired properties, such as being unit free [7], correcting for chance agreement [8] or being unaffected by the order of the observers [9]. Confusion between reliability and agreement may arise because some statistical measures originally developed to summarize agreement in a sample, such as the quadratic kappa coefficient [10], were later shown to estimate reliability [11, 12].

Second, while guidelines for reporting reliability and agreement studies are generally followed (e.g., GRRAS guidelines [13]), the choice of specific statistical measures was almost never justified and sometimes not in line with the aim of the study (e.g., agreement measures were used to assess reliability). Furthermore, when more than two observers were involved in the study, it was not always clear how an overall statistical measure was obtained [3]. Third, the value of agreement and reliability coefficients was interpreted independently of the statistical measure used following fixed subjective guidelines (e.g., [14]), while confidence intervals were seldom reported. When a confidence interval was reported, the method used to compute it was not always clear. Finally, the sample size (number of observers and number of patients) was almost never justified by a sample size calculation.

This paper therefore aims to review the most common measures of agreement and reliability when two or more observers classify patients/objects on an ordinal scale (see Vanbelle S, Hernandez Engelhart C, Blix E, Agreement and reliability on binary scales in obstetrics (submitted) for binary scales). It will distinguish between the concepts of agreement and reliability, provide a clear interpretation of the most common statistical measures, expose an appropriate statistical inference procedure and set up a new sample size determination procedure to plan agreement and reliability studies.

## Methods

This paper focuses on ordinal scales. Ordinal scales, such as CTG classification (normal/suspicious/pathological), possess at least two specific characteristics that should be considered in the statistical analysis. First, the scale is bounded and finite. In the CTG example, the classification can only take three different values. Second, although the categories are ordered, numbers attributed to the different categories are merely labels, meaning that defining a distance between categories is arbitrary. A common assumption is to consider that adjacent categories are equidistant. Methods developed for ordinal scales are therefore different from those developed for quantitative scales, although, as we will see, there is sometimes a correspondence between the two.

Historically, agreement measures were defined in terms of *estimators*, i.e., the formula used to calculate an estimate based on a sample, rather than in terms of *population parameters*, which represent quantities that characterize a population. While estimators are presented in the main manuscript, the related population parameters are given in supplementary file A for agreement and supplementary file B for reliability.

### Difference between intra- and inter- agreement/reliability studies

Two different types of studies are common when assessing either agreement or reliability, namely, intra- and inter-observer studies. In intra-observer studies, replicated measurements of the same patients/objects are made by one observer under identical conditions. That is, only the time at which the measurements are made differs between the replicates. In that case, it is frequently assumed that the order of the measurements does not affect the results (i.e., the *interchangeable ratings assumption* [15]). For example, suppose that two patients are evaluated two times by the same observer on a 3-ordinal scale. The interchangeable ratings assumption means that swapping the replicated observations within some of the patients/objects will not change the value of the agreement or reliability coefficient. For example, imagine that Patient 1 has the observations “1” and “2” for the first and second time points, whereas Patient 2 has the observations “2” and “3”, respectively. Swapping the observations of Patient 1, that is considering that “1” is the observation obtained at the second time point and that “2” is obtained at the first time point, will not affect the value of the statistical measure.

Inter-observer studies refer to studies where replicated measurements of the same patients/objects are made by different observers under identical conditions. The interchangeability assumption is often not appropriate because it assumes that all observers have the same rating style. Statistical measures accounting for systematic differences in the rating style of the observers (e.g., some observers systematically assess more CTGs as suspicious or pathological than others do) were therefore developed.

Sometimes, patients are assessed several times by the same set of observers. For example, the same 3 observers can assess the same CTGs two times. This permits the evaluation of both intra- and inter-observer agreement/reliability in one study. In that case, statistical techniques to quantify the agreement/reliability and corresponding confidence intervals are more complex because of the presence of different observers and replicated measurements made by one observer at the same time (e.g., [16]). Nevertheless, it is possible to use the statistical measures presented in this paper by selecting particular assessments. For instance, intra-observer agreement/reliability can be evaluated separately for each observer and the inter-observer agreement/reliability can be reported at each time point. Reporting the intra-observer agreement/reliability for each observer and the inter-observer agreement/agreement at each time point has the advantage of allowing the use of common statistical software to construct confidence intervals. However, this approach results in a loss of information, leading to wider confidence intervals. Note that if the interchangeability assumption is satisfied (i.e., similar inter-observer agreement/reliability levels are observed across all time points), one overall inter-observer agreement/reliability measure can be reported by randomly selecting one assessment time for each observer.

### Agreement measures between two observers

Consider the study of [17] investigating the inter-observer agreement in expert interpretation of CTG tracings following the guidelines of the Federation of Gynecology and Obstetrics [18]. Thirty-three CTGs were classified as normal (1), suspicious (2) or pathological (3) by three experts. Only the observations made by the Experts 1 and 2 are used to illustrate this section.

The classification of *N* patients or objects (e.g. CTGs) by two observers on an ordinal scale with *K* categories (e.g., “normal”, “suspicious” and “pathological”) can be summarized in a $K\times K$ classification table (Table 1) in terms of counts ($n_{jk}$) or proportions ($p{_{jk}}$) ($j,k=1,\cdots ,K$).

**Table 1:** Classification of *N* patients or objects by two observers on a *K*-ordinal scale in terms of counts (proportions)

	Observer 2					
Observer 1	1	$\ldots$	j	$\ldots$	K	Total
1	$n_{11}\ (p_{11})$	$\cdots$	$n_{1j}\ (p_{1j})$	$\ldots$	$n_{1K}\ (p_{1K})$	$n_{1.}\ (p_{1.})$
$\vdots$	$\vdots$		$\vdots$	$\vdots$		
j	$n_{j1}\ (p_{j1})$	$\cdots$	$n_{jj}\ (p_{jj})$	$\ldots$	$n_{jK}\ (p_{jK})$	$n_{j.}\ (p_{j.})$
$\vdots$	$\vdots$		$\vdots$	$\vdots$		
K	$n_{K1}\ (p_{K1})$	$\cdots$	$n_{Kj}\ (p_{Kj})$	$\ldots$	$n_{KK}\ (p_{KK})$	$n_{K.}\ (p_{K.})$
Total	$n_{.1}\ (p_{.1})$	$\cdots$	$n_{.j}\ (p_{.j})$	$\ldots$	$n_{.K}\ (p_{.K})$	*N* (1)

$n_{11}$: number of objects classified in category 1 by both observers

$n_{1j}$: number of objects classified in category 1 by observer 1 and category *j* by observer 2

$n_{j1}$: number of objects classified in category *j* by observer 1 and category 1 by observer 2

$n_{KK}$: number of objects classified in category *K* by both observers

$n_{1.}=n_{11}+\cdots +n_{1K}$: number of objects classified in category 1 by observer 1

$n_{j.}=n_{j1}+\cdots +n_{jK}$: number of objects classified in category *j* by observer 1

$n_{.1}=n_{11}+\cdots +n_{K1}$: number of objects in category 1 by observer 2

$n_{.j}=n_{1j}+\cdots +n_{Kj}$: number of objects classified in category *j* by observer 2

$n=n_{11}+n_{12}+\cdots +n_{(K-1)K}+n_{KK}$: total number of objects

**Fig. 1:**
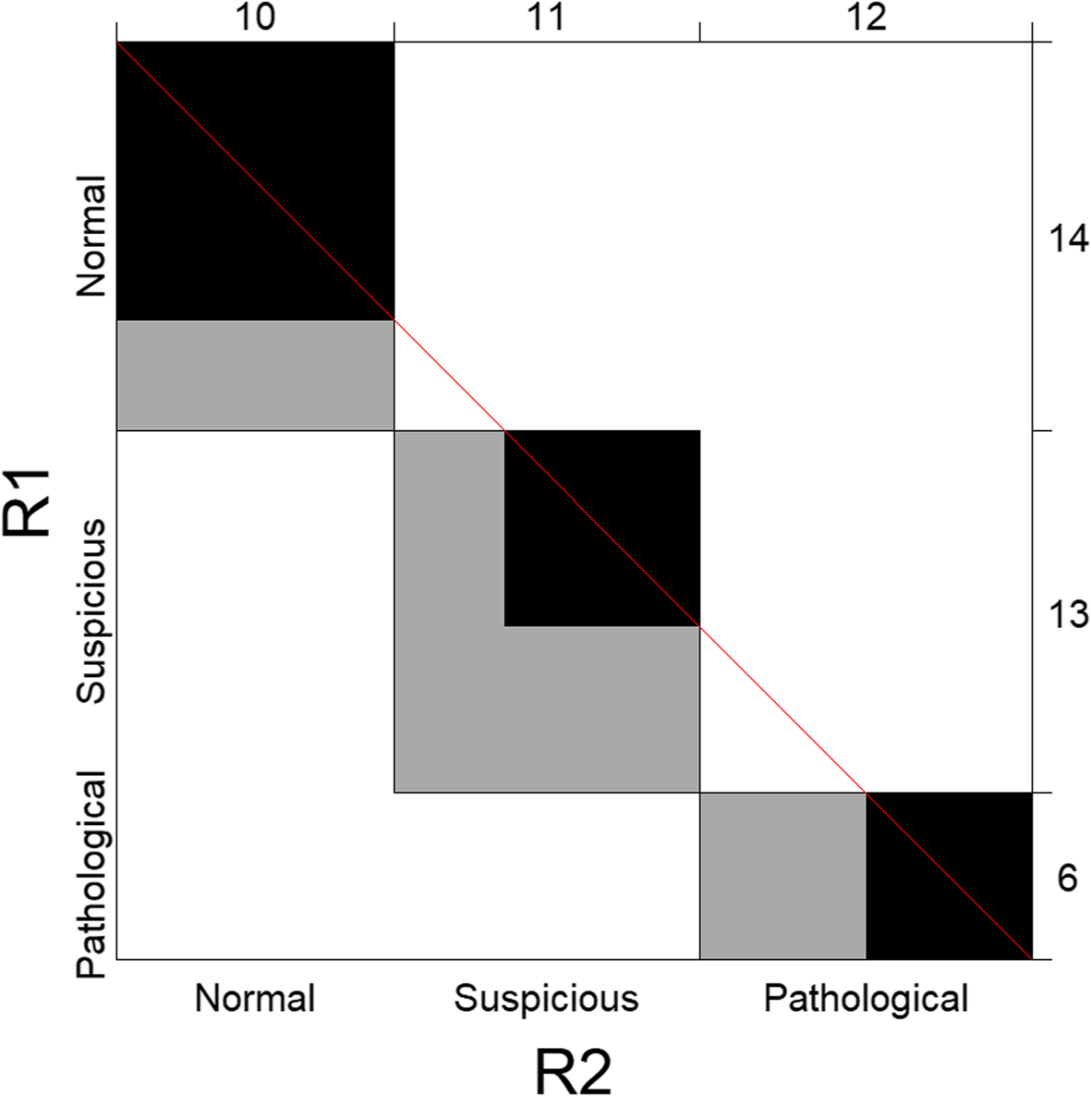
CTG example. Agreement plot

Considering the CTG example, we obtain Table 2. It is also possible to summarize this classification graphically, using an agreement plot [19] (see Fig. 1).

**Table 2:** CTG example

	Expert 2			
Expert 1	1	2	3	Total
1	10 (0.30)	4 (0.12)	0 (0.00)	14 (0.42)
2	0 (0.00)	7 (0.21)	6 (0.18)	13 (0.39)
3	0 (0.00)	0 (0.00)	6 (0.18)	6 (0.18)
Total	10 (0.30)	11 (0.33)	12 (0.36)	33 (1)

Classification of 33 CTGs by Experts 1 and 2 as “normal (1)”, “suspicious (2)” and “pathological (3)” in terms of counts (proportions)

In the agreement plot, every rectangle represents one cell of the classification table. The size of each rectangle is determined by the corresponding row and column totals. If the corners of the rectangles are below (above) the red line, Expert 2 gives higher (lower) scores on the ordinal scale than Expert 1 does. In the CTG example, “normal” is associated with the lowest score of 1, and “pathological” is associated with the largest score of 3. Therefore, if the corners of the rectangles are below the red line, Expert 2 gives higher scores (assessing more of the CTG tracings as suspicious and abnormal) than Expert 1 does. If the rectangles are above the red line, it means that Expert 2 gives lower scores (assessing more tracings as normal) than Expert 1. The rectangles are shaded gray. The black area is proportional to the counts in the diagonal cells of the table. Then, the further a cell is from the diagonal, the lighter the gray color is. In the CTG example, there are only observations at a distance of one cell from the diagonal. They disagreed on whether a CTG tracing was normal/suspicious, or suspicious/pathological. There were no cases with disagreements over two categories, where the same tracing was interpreted as both normal and pathological. Furthermore, Expert 2 scored CTGs more often as suspicious or pathological than Expert 1 did because the corners of the rectangles are below the diagonal line. Most disagreement occurs for the categories “suspicious” and “pathological” where the gray area is large compared with the black area.

The simplest agreement measure is the sum of the diagonal elements of the classification table, called *the proportion of agreement* and denoted by $p_o$,

1 $$\begin{aligned} p_o=\sum _{k=1}^{K}\frac{n_{kk}}{N}=\sum _{k=1}^{K}p_{kk}. \end{aligned}$$

The proportion of disagreement is equal to $q_o=1-p_o$. In the CTG example, Experts 1 and 2 agree on (10+7+6)/33=69.7% of the CTG traces. Therefore, they disagree on 100-69.7=31.3% of the CTG traces.

All disagreements are considered equally in $p_o$. In some circumstances, disagreements one category apart can be seen as partial agreement and less serious than disagreements two or more categories apart. For example, in the CTG example, disagreements between the categories “suspicious” and “normal” or “suspicious” and “pathological” can be considered less serious than the disagreement between “normal” and “pathological”. Disagreement (or agreement) weights can then be allocated to every cell of the classification table to reflect it. Disagreement weights are usually symmetric and increase proportionally with the distance (i.e., number of categories) between the ratings. Disagreement weights therefore increase when cells are further away from the diagonal of Table 1.

Disagreement weights can be scaled to take values between 0 and 1 with a value of 0 in the cells representing agreement (diagonal cells) and the maximum value of 1 in the cells with the maximum distance between the ratings of the two observers (i.e., when the two ratings are $K-1$ categories apart). Two common weighting schemes are the linear [20] and the quadratic [12] weighting schemes. While they were introduced subjectively on an ad-hoc basis, the resulting coefficients turn out to have a simple and straightforward interpretation. Disagreement weights increase linearly (quadratically) with the distance between the ratings for the linear (quadratic) weighting scheme. For example, with 3 categories as in the CTG example, the linear disagreement weights are equal to 0, 1 and 2 when the two ratings are 0, 1, or 2 categories apart (or weights are equal to 0, 1/2 and 1 for the scaled version). The quadratic weights are equal to 0, 1 and 4 (or 0, 1/4, 1 for the scaled version) (see supplementary file A for the formulas).

The *weighted disagreement* is then the weighted sum of all the elements in Table 1, i.e.,

2 $$\begin{aligned} q_{ow}=\sum _{j=1}^{K}\sum _{k=1}^{K}v_{jk}p_{jk} \end{aligned}$$

where $v_{jk}$ are the disagreement weights. Note that if the unscaled disagreement weights are used, $q_{ow}$ can be larger than 1. This is why it is not called the “weighted proportion of disagreement”. When scaled nominal disagreement weights are used (weights of 1 in all cells, except on the diagonal where weights are equal to 0), the *proportion of disagreement* is obtained,

3 $$\begin{aligned} q_o=1-\sum _{j=1}^{K}p_{jj}=1-p_o. \end{aligned}$$

Using the unscaled linear disagreement weights, the resulting coefficient is also known as *the mean absolute deviation*,

4 $$\begin{aligned} \widehat{MAD}=\sum _{j=1}^{K}\sum _{k=1}^{K}v_{jk}p_{jk}=\sum _{j=1}^{K}\sum _{k=1}^{K}|j-k|p_{jk}=\frac{1}{N}\sum _{i=1}^{N}|y_{i1}-y_{i2}| \end{aligned}$$

where $y_{i1}$ (resp. $y_{i2}$) denotes the category chosen by observer 1 (resp. 2) for patient/object $i=1,\cdots ,N$. The mean absolute deviation is the mean number of categories between the ratings made by the two observers [21]. On the other hand, considering unscaled quadratic weights will result in a coefficient also known as *the mean squared deviation*

5 $$\begin{aligned} \widehat{MSD}=\sum _{j=1}^{K}\sum _{k=1}^{K}v_{jk}p_{jk}=\sum _{j=1}^{K}\sum _{k=1}^{K}(j-k)^2p_{jk}=\frac{1}{N}\sum _{i=1}^{N}(y_{i1}-y_{i2})^2 \end{aligned}$$

that is, the mean squared number of categories between the ratings made by the two observers. In the CTG example, we have $\widehat{MAD}=(0\times23+1\times10+2\times0)/33=0.30$. This means that, on average, the classification made by Experts 1 and 2 differs by 0.30 categories. We also have $\widehat{MSD}=(0\times23+1\times10+4\times0)/33=0.30$. This means that, on average, the squared number of categories between the classification made by Experts 1 and 2 differ by 0.30 category. While the interpretation of *MSD* is less intuitive than of *MAD*, it possesses better mathematical properties (e.g., when taking a derivative) and is therefore commonly used.

These coefficients were criticized because they do not account for the fact that some agreements are only expected by chance, i.e., expected if the two observers classify the patients/objects randomly [8]. The idea of [8, 10], who introduced the kappa coefficients, was therefore to compare the disagreement observed to the disagreement expected by chance. Several definitions of chance have been introduced over the years (e.g., [8, 15, 22]). Three common definitions of chance disagreement related to each other, are given below. Chance disagreement is the disagreement obtained when each observer is tossing a dice with *K* sides that

1. is fair, i.e., has a probability of 1/*K* of landing on every side.
2. is unfair, with the probability for the dice to land on side *k* equal to the proportion of objects classified in category *k* by that observer. As a result, the two observers use different unfair dices.
3. is unfair, with a probability for the dice to land on side *k* equal to the overall proportion of objects classified in category *k*. This results in the two observers using the same unfair dice.

Weighted kappa coefficients are obtained by comparing the weighted disagreement observed in the data to the weighted disagreement expected by chance. The exact formulas are given in supplementary file A. The value of these weighted kappas vary between −1 and 1. A value of 1 means perfect agreement (all observations are on the diagonal cells of Table 1), a value of 0 means that weighted agreement is not better than chance, and negative values indicate that the weighted agreement is smaller than the one expected by chance. Note that a negative weighted kappa may indicate that one of the observers potentially misinterpreted the scale, for example by considering the scale in the opposite way.

In this paper, three weighting schemes and three chance definitions are presented, leading to nine possible combinations. Among these nine combinations, only some, to the best of our knowledge, are commonly used, implemented in standard software and studied in the literature (see Table 3 for names and references).

**Table 3:** Common weighted agreement coefficients in the case of two observers

Chance definition	Nominal	Linear	Quadratic
1	PABAK [23], G index [24]	no specific name	no specific name
	free marginal kappa [25]		
	Brennan-Prediger kappa [25]	Brennan-Prediger weighted kappa [22]	
2	Cohen’s kappa[8]	Linear weighted kappa[10]	Quadratic weighted kappa [10]
		Chicchetti-Alisson weighted kappa [26]	Fleiss-Cohen weighted kappa [12]
			concordance correlation coefficient [7]
3	Intraclass kappa[15],	no specific name	no specific name
	Scott’s pi [9]		

Note that under chance definition 1, the obtained weighted kappa coefficients are simple functions of $q_{ow}$. For example, when nominal weights are used, $\hat{\kappa }_{w1}=1-2q_{ow}$. This could explain why little attention has been given to these coefficients in the literature, as $q_{ow}$ is simpler to interpret. The combination of weights and chance definition 3 is less commonly used for ordinal scales although the statistical properties of these coefficients have been studied in the literature [27–31]. One reason could be that these coefficients are not available in common menu-driven statistical software.

In the CTG example, the weighted kappa coefficients between Experts 1 and 2 using the different weighting schemes and the different chance definitions are given in Table 4.

**Table 4:** CTG example

Chance definition	Nominal	Linear	Quadratic
1 (Completely random)	0.55	0.66	0.77
2 (Different)	0.55	0.66	0.77
3 (Same)	0.54	0.65	0.76

Weighted kappa coefficients using different weighting schemes and chance definitions

When considering a particular weighting scheme, the inequality $\hat{\kappa }_{w1}>\hat{\kappa }_{w2}> \hat{\kappa }_{w3}$ generally holds. The coefficients under chance definitions 2 and 3 coincide when the two observers classify the same proportion of patients or objects in the *K* categories. If this proportion is further equal to 1/*K*, the coefficients obtained under the three chance definitions are equal. Therefore, a difference between $\hat{\kappa }_{w2}$ and $\hat{\kappa }_{w3}$ is a sign that the proportion of patients/objects in the *K* categories differs between the two observers and between $\hat{\kappa }_{w1}$ and $\hat{\kappa }_{w2}$ that the proportion of patients/objects in the *K* categories is not equal to 1/*K*. The inequality $\hat{\kappa }_{w2}<\hat{\kappa }_{w3}$ holds in very particular circumstances. The inequality will occur when the proportion of patients in the categories of the scale differs markedly form 1/*K* and categories where one observer classified the largest number of patients are the categories where the other observer classified the smallest number of patients. There is no general rule ordering the weighted kappa coefficients obtained with the different weighting schemes [31–33], but the linear weighted kappa is often smaller than the quadratic weighted kappa. Here, the proportion of disagreement ($q_o=0.31$) is approximately 0.45 times the proportion expected by chance, whereas the mean distance between the two classifications ($\widehat{MAD}=0.15$) is approximately 0.34 times the mean distance expected by chance.

### Agreement between more than two observers

Agreement can be extended to more than two observers in several ways. For example, we can say that there is agreement between three observers when at least two observers agree (majority agreement), when they all agree (total consensus agreement) or we can compute a weighted mean agreement over all pairs of observers. The last generalization is the most popular when nominal weights are used. Using linear and quadratic weights, this generalization was also considered by [27–30, 34] under chance definitions 2 and 3, whereas total consensus agreement was considered by [30, 35]. While total consensus agreement is the most restrictive definition and pairwise agreement the least restrictive one, it is possible to define *g*-wise agreement by considering agreement between *g*-uples of observers ($g=2,\cdots ,R$) [35, 36]. Here too, the resulting agreement coefficients do not possess specific names, are not often used, and are not available in common menu-driven statistical software.

To describe the disagreement pattern among the *R* observers, the data can be summarized in an overall $K\times K$ symmetric table obtained by taking the average over all pairs of observers (see Table 5 for the CTG example).

**Table 5:** CTG example

	Observer 2 of the pair		
Observer 1 of the pair	Normal (1)	suspicious (2)	Pathological (3)
Normal (1)	0.26	0.08	0.00
suspicious (2)	0.08	0.25	0.09
Pathological (3)	0.0	0.09	0.14

$K\times K$ table summarizing the classification made by the 3 observers

We can observe in Table 5 that the amount of disagreement between the categories ’suspicious’ and ’normal’ is similar to the amount of disagreement between the categories ’suspicious’ and ’pathological’. Note that it is not possible to compute weighted kappa coefficients under chance definition 2 based on this table. This table is only intended to summarize the overall disagreement pattern.

The formulas for the weighted disagreement, the expected weighted disagreement and the weighted kappa coefficients are given in Supplementary file A when considering the pairwise definitions [27–30, 34]. In the presence of only two observers ($R=2$), all these weighted coefficients reduce to the coefficients defined in Section Agreement between more than two observers.

In the CTG example, we have, for the three experts, $p_o=0.66$, $\widehat{MAD}=0.34$ and $\widehat{MSD}=0.34$. The weighted kappa coefficients are given in Table 6.

**Table 6:** CTG example

Chance definition	Nominal	Linear	Quadratic
1 (completely random)	0.48	0.60	0.72
2 (Different)	0.48	0.58	0.70
3 (Same)	0.47	0.57	0.70

Weighted kappa coefficient among the three experts

This means that, on average, two experts agree on the classification of 66% of the CTGs. Furthermore, there is, on average, a difference of 0.34 categories between the classifications made by two experts, which is approximately 0.40 times the weighted disagreement expected by chance.

### Reliability versus agreement

Reliability is defined as the ability of a measurement instrument to distinguish between patients or objects in a given population. Reliability is defined as the proportion of variability due to differences between the patients or objects rather than measurement error under specific mathematical models depending on the study design [2]. We adopt the definition of reliability as an intraclass correlation between replicated measurements (see supplementary file B for formal definitions and mathematical assumptions). Intraclass correlations vary between 0 and 1. Values close to 1 indicate that most of the variability in the observations can be explained by the fact that patients/objects are different rather than resulting from measurement error. Small values indicate that the measurement error is important as compared to the variability between patients/objects. The same amount of measurement error will appear more important when compared to the variability between patients in homogeneous populations of patients than in heterogeneous populations. Therefore, reliability coefficients depend on the homogeneity of the population of patients and can be low in homogeneous populations. When reliability is estimated on a sample, negative values can be obtained, depending on the estimation method used.

When quadratic weights and chance definitions 2 or 3 are used, several authors (e.g., [12, 37, 38]) have shown that the resulting weighted kappa coefficients are equivalent to the estimator of an intraclass correlation, provided that the number of patients or objects *N* is greater than 20 [37].

Using chance definition 2, $\hat{\kappa }_{w2}$ was shown to estimate the intraclass correlation coefficient for agreement under a two-way ANOVA model. The two-way ANOVA model is a mathematical model usually used in inter-observer studies where the same set of observers rates a sample of objects/patients. The intraclass correlation coefficient for agreement accounts for possible differences in the rating styles of the observers.

Under chance definition 3, $\hat{\kappa }_{w3}$ was shown to estimate the intraclass correlation defined under a one-way ANOVA model. A one-way ANOVA model is used in inter-observer studies where each object/patient is rated by a different set of observers because it is not possible to disantangle the effect of the observers and the error in that case or in intra-observer studies. Specifically, this mathematical model assumes that we cannot distinguish between the ratings within an object/patient, e.g., swapping ratings within some patients will not affect the value of the coefficient.

Because these specific weighted kappa coefficients possess the same properties as reliability coefficients do, weighted kappa coefficients can be close to 0 (e.g., $\hat{\kappa }_{w3} \sim 0$) despite good weighted agreement (e.g., $p_{ow}=0.85$), especially in homogeneous populations. This provides a simple explanation to what is know as a kappa paradox [26], i.e., that kappa coefficients depend on the probability for the patients to be classified in the categories of the scale.

The motivating example is an inter-rater agreement study where the same set of observers rates a sample of objects/patients. Therefore, definition 2 of chance agreement is preferred over definition 3 to account for possible differences in the rating styles of the observers i.e., possible systematic differences between the observers. The obtained quadratic weighted kappa coefficient, $\hat{\kappa }_{w2}=0.70$ is therefore an estimate of the intraclass correlation for agreement in a two-way ANOVA model and can be interpreted as follows. Approximately 70% of the total variability in the CTG classifications can be attributed to differences between the patients for whom CTGs were provided rather than other sources namely, systematic differences between the observers and other sources of measurement error (see Equation B.10 in supplementary file B).

### Statistical inference

It is important to determine confidence intervals around the agreement and reliability coefficients because these coefficients were evaluated on a sample of patients/objects rated by a sample of observers. Different samples will therefore lead to different values of the agreement and reliability coefficients.

Several methods can be used to make statistical inference for $p_{ow}$, $q_{ow}$, $\widehat{MAD}$, $\widehat{MSD}$ and the weighted kappa coefficients. Here, we construct a two-sided ($1-\alpha )\times$ 100% Wald confidence interval,

6 $$\begin{aligned} statistic\pm Q_z(1-\alpha /2)SE(statistic) \end{aligned}$$

where *statistic* is $p_{ow}$, $q_{ow}$, $\widehat{MAD}$, $\widehat{MSD}$ or a weighted kappa coefficient and where $Q_z(1-\alpha /2)$ is the ($1-\alpha )\times$ 100% percentile of the standard normal distribution. We present the formula for *SE*(*statistic*) derived with the delta method for all coefficients presented in this paper in supplementary file C. This method was chosen because it can be applied to all these coefficients and presents good statistical properties in general. The statistical properties of the confidence intervals for weighted kappa coefficients can be improved by considering Fisher-Z transform, which is known for being a variance stabilizing function. Formulas considering this transform are also given in supplementary file C.

In the CTG example, we obtain the following confidence intervals. For the proportion of agreement, we have (0.49,0.82); for the proportion of disagreement, we have (0.18,0.50); for $\widehat{MAD}$, we have (0.18,0.50); and for $\widehat{MSD}$, we have (0.18,0.50). The three last statistical measures and confidence intervals coincide because the table is tridiagonal, i.e. there are only observations in the diagonals cells and the cells next to the diagonal. The confidence intervals for the various kappa coefficients are given in Table 7 with and without the Fisher Z-transform for comparative purposes.

**Table 7:** CTG example. Weighted kappa coefficient among the three experts

Chance definition	Nominal	Linear	Quadratic
Wald confidence interval			
1 (completely random)	0.48 (0.31,0.66)	0.60 (0.46, 0.74)	0.72 (0.61, 0.84)
2 (Different)	0.48 (0.31,0.65)	0.58 (0.43, 0.73)	0.70 (0.58, 0.82)
3 (Same)	0.47 (0.29,0.65)	0.57 (0.42, 0.73)	0.70 (0.57, 0.82)
Fisher Z-transform confidence interval			
1 (completely random)	0.48 (0.32,0.62)	0.60 (0.47, 0.70)	0.72 (0.64, 0.79)
2 (Different)	0.48 (0.32,0.61)	0.58 (0.45, 0.69)	0.70 (0.60, 0.77)
3 (Same)	0.47 (0.30,0.61)	0.57 (0.44, 0.69)	0.70 (0.60, 0.77)

We can observe that the confidence intervals for all agreement measures are wide. For example, on average, we are 95% confident that two experts agree on between 49% and 82% of the CTGs. The amount of uncertainty can be limited by selecting an appropriate number of observers and/or patients, as we will see the next section.

### Sample size calculation

In the planning stage of an agreement/reliability study, the number of observers and patients (or objects) should be determined to be able to test statistical hypotheses or to estimate agreement/reliability with a certain precision. In both cases, the most frequent situation is to determine the minimum number of patients needed given the number of observers. The calculations involve the formula *SE*(*statistic*) (see Eq. 6). Since the formulas for the various agreement/reliability coefficient are complex (see supplementary file C) and almost always require the researcher to give an idea of the value expected in all cells of the contingency table, no analytical formula is currently available. We propose a simple but time-consuming procedure to obtain an order of magnitude of the minimum number of objects/patients needed to reach a certain criterion, on the basis of a confidence interval or a statistical hypothesis test.

We propose simulating data using the R package orddata [39] given the following information: the proportion of patients expected in each of the *K* categories of the scale for all the observers, the expected agreement level, the number of observers and the number of objects/patients. By doing so, we assume that all observers have the same propensity to classify patients or objects in these *K* categories, that is, we do sample size calculations under chance definition 3. With that information, several classification tables are possible. When we perform one simulation, we obtain one specific contingency table that we name a scenario. We repeat this process a large number of times (e.g., 1000) and determine the proportion of simulated datasets satisfying a specific criterion. This will provide an idea of the number of objects/patients needed in agreement and reliability studies to satisfy a criterion such as a reasonable confidence interval width or sufficient power to test statistical hypotheses.

### Confidence interval approach

In the confidence interval approach, we aim to achieve a confidence interval with a width smaller than a predefined value *w* around the agreement/reliability level. Using the Wald confidence interval, the width of the confidence interval is given by $2Q_z(1-\alpha )SE(statistic)$ (see Eq. 6) where the form of $SE(statistic)$ differs, depending on the agreement/reliability measure considered (see supplementary file C). The minimal number of patients or objects *N* needed to obtain a width smaller than or equal to *w* is obtained by solving this equation for *N*. For weighted kappa coefficients, Fisher-Z transform is further considered. As stated before, it is hardly possible to obtain any analytical formula in the case of ordinal scales. Suppose that we would like to plan a study similar to the CTG example, where the ordinal scale has 3 categories. Further assume that it is possible to recruit between 5 and 8 observers, and that the researchers would like to achieve a width of the confidence interval smaller than 0.20 around a planned quadratic weighted kappa of 0.75. As a random sample of high-risk patients in the clinic is planned to be used, they expect 40% of normal cases, 40% of suspicious cases and 20% of pathological cases. The percentage of simulated datasets satisfying the criterion (a width smaller than 0.20) and the maximum width of the confidence intervals obtained on the simulated data are given in Table 8 for different numbers of CTGs.

**Table 8:** Percentage of the 1000 simulated datasets achieving the criterion of a 95% confidence interval width of 0.20 or less (considering Fisher-Z transform) around a quadratic weighted kappa coefficient of 0.75 when expecting a proportion of normal, suspicious and pathological cases of 0.40, 0.40 and 0.20, respectively

Number of CTGs	30	40	50	60
Number of observers				
5	80.6 (0.35)	94.6 (0.30)	98.9 (0.25)	99.7 (0.23)
6	85.3 (0.33)	97.6 (0.24)	99.7 (0.24)	100 (0.17)
7	85.8 (0.30)	98.5 (0.26)	99.8 (0.22)	100 (0.17)
8	89.5 (0.31)	98.8 (0.26)	100 (0.20)	100 (0.17)

The maximum width of the confidence interval obtained on the simulated datasets is indicated in brackets

To determine the most realistic combination of the number of observers and patients, we should account for the fact that some observers and some patients can withdraw from the study. Therefore, given what is possible in practice and the results of the simulation study, a total of 6 observers and 60 patients are planned for the study.

### Testing approach

In the testing approach, we are interested in testing statistical hypotheses of the form

$$\begin{aligned} H_0: \rho \le \rho _0 \text{ vs } H_1: \rho> \rho _0, \text{ i.e. } \rho = \rho _A, \end{aligned}$$

where $\rho _0$ is the agreement/reliability level under the null hypothesis and $\rho _A$ is the agreement/reliability level under the alternative hypothesis with a pre-specified power of $1-\beta$ and a type-one error $\alpha$. To find the minimal number of patients needed, we have to solve the following equation for *N*,

$$\begin{aligned} \Phi _z\left( \frac{\rho _A-\rho _0}{\sqrt{var(\rho _A)}}-Q_z(1-\alpha )\right) \ge 1-\beta \end{aligned}$$

where $\Phi (.)$ is the cumulative normal distribution function and $\sqrt{var(\rho _A)}$ is the standard error of the selected agreement/reliability coefficient under the alternative hypothesis. It is not possible to solve this equation analytically most of the time. Again, we will simulate data to obtain an idea of the percentage of simulated datasets that reaches the criterion (see Table 9).

**Table 9:** Percentage of the 1000 simulated datasets with an empirical power of at least $1-\beta =0.80$ to test the statistical hypotheses about the quadratic weighted kappa coefficient with $\rho _0=0.70$, $\rho _A=0.80$ and $\alpha =0.05$ and when expecting a proportion of normal, suspicious and pathological cases of 0.4, 0.4 and 0.2, respectively

Number of CTGs	30	40	50	60	70	80
Number of observers						
6	9.2 (0.00)	25.2 (0.38)	52.2 (0.43)	77.0 (0.54)	94.4 (0.63)	97.6 (0.73)
7	10.4 (0.26)	31.6 (0.38)	57.6 (0.50)	80.2 (0.60)	95.0 (0.67)	99.2 (0.73)
8	12.2 (0.33)	32.6 (0.45)	62.6 (0.57)	85.4 (0.65)	96.2 (0.74)	99.6 (0.77)

The minimum power calculated given the 1000 simulated datasets is indicated in brackets

Here too, accounting for what is most convenient in practice and the possible drop out of observers and patients, the study is planned with 7 observers and 70 patients.

### Statistical software

A summary of the possibilities existing in the main software is presented in Table 10. In SPSS, it is possible to compute the linear and quadratic kappa for two observers under the menu scale, weighted kappa. In STATA, this is possible by using the functions kap and kappa (however the standard error is only valid under the null hypothesis that the agreement coefficient is equal to 0 and should not be used to compute a confidence interval in general). In R, agreement coefficients are available in the *VCD*, *psych*, *irr*, *irrCAC* and *magree* packages. Note that chance definition 3 is only available in *magree* and *irrCAC* packages. No software allows sample size calculations for the coefficients presented in this paper.

**Table 10:** Statistical software. Agreement and reliability measures

Statistical software	SPSS	STATA	SAS	R
$\hat{\kappa }_{w2}$,$\hat{\kappa }_{w3}$(2 observers)	scale	kap^a^	PROC	irr^a^, psych, vcd,
		kapci^b^	FREQ	irrCAC, magree
$\hat{\kappa }_{w2}$,$\hat{\kappa }_{w3}$(2 or more observers)	not available			magree
				irrCAC
*MAD*,*MSD*,$\hat{\kappa }_{w1}$(2 observers)	not available	matrix dissimilarity^c^	PROC DISTANCE^c ^	catsim^c^
				irrCAC

^a^ the standard error is not valid in general but only to test the statistical hypothesis$\kappa =0$

^b^ Bootstrap confidence interval

^c^ only quadratic weights

An R statistical package *weightagree* available on Github (https://github.com/svanbelle) and a Shiny app (https://svanbelle.shinyapps.io/weightagree/) were developed to determine the confidence intervals of the various weighted agreement and disagreement coefficients using the delta method and perform sample size calculations.

While the package *irrCAC* also permits to compute the weighted agreement coefficients presented in this paper, the standard errors are derived with a linearization method rather than the delta method. Furthermore, our package also includes confidence intervals based on Fisher-Z transform and sample size calculations.

### General recommendations

It is recommended to report the distribution of patients on the ordinal scale, as the weighted kappa coefficients depend on this distribution. In agreement studies, we suggest reporting at least one unscaled and one scaled coefficient. This is because scaled coefficients compare the weighted disagreement to what would be expected by chance but do not provide the magnitude of the weighted disagreement itself.

For intra- and inter-observer agreement studies, we recommend the proportion of agreement or the mean absolute deviation with a 95% Wald confidence interval as unscaled agreement measure because they possess the simplest interpretation. As scaled agreement coefficient, we recommend using the linear weighted kappa coefficient with chance definition 2 for inter-observer studies and chance definition 3 for intra-observer studies.

In reliability studies, the quadratic weighted kappa coefficient is advised, again with chance definition 2 for inter-observer studies and chance definition 3 for intra-observer studies. For all weighted kappa coefficients, it is advised to use confidence intervals based on Fisher Z-transform.

Below is a summary of the key coefficients and their interpretation:,

$p_{o}$ (proportion of agreement): Mean proportion of agreement between two observers

$\widehat{MAD}$ (mean absolute deviation): Mean distance between the ratings of any pair of observers

$\hat{\kappa }_{w2}$ (linear weights): Observed *MAD* is $1-\hat{\kappa }_{w2}$ times $MAD$ expected by chance, accounting for the rating style of each observer

$\hat{\kappa }_{w3}$ (linear weights): Observed *MAD* is $1-\hat{\kappa }_{w3}$ times $MAD$ expected by chance, assuming the same rating style for all observers, i.e., that the observers are interchangeable

$\hat{\kappa }_{w2}$ (quadratic weights): Observed MSD is $1-\hat{\kappa }_{w2}$ times $MSD$ expected by chance, accounting for the rating style of each observer. In terms of reliability: proportion of total variance that can be attributed to differences between patients/objects rather than systematic difference between the observers and other sources of measurement error

$\hat{\kappa }_{w3}$ (quadratic weights): Observed MSD is $1-\hat{\kappa }_{w3}$ times $MSD$ expected by chance, assuming the same rating style for all observers, i.e., that the observers are interchangeable. In terms of reliability: proportion of total variance that can be attributed to differences between patients/objects rather than differences within patients/objects (i.e., between the replicates)

## Discussion

We reviewed the main agreement and disagreement measures for ordinal scales. Importantly, we intended to provide a straightforward interpretation of the coefficients. Like other authors [13], we do not advise the use of standard tables to qualify the strength of agreement/reliability as poor, fair, etc. (see e.g. the well-known Landis and Koch table [14] for kappa coefficients) for at least three reasons. First, this classification is subjective. The interpretation and qualification of agreement/reliability levels should be adapted to the context in which the measurement instrument will be used. For example, an agreement/reliability level of 0.70 between observers could be considered as satisfactory in the context of heart rate evaluation of recreational runners after a race but not in the medical context. In a medical context, when interpreting CTGs, an agreement/reliability level of 0.70 may lead to both under- and over-treatment. Moreover, in psychology, where some constructs are difficult to evaluate (e.g., intelligence), researchers could be satisfied with lower agreement/reliability levels than in some medical context where more objective measurements are made (e.g., measuring the degree of a burn). The authors nevertheless acknowledge that different classification rules will also be subjective. It is nevertheless important to account for the context and provide a rationale for the classification rules used. Second, this kind of classification table does not consider the uncertainty resulting from taking only a sample of observers/patients, e.g., the confidence interval. An agreement/reliability level of 0.71 would be considered as “substantial”, with a lower bound of the confidence interval of 0.69 or 0.49. The authors advise researchers to account for confidence interval bounds when interpreting agreement/reliability coefficients. For example, a range of values based on a confidence interval can be reported instead of only the agreement/reliability estimate. Finally, this type of table is often referred to in the literature independently of the statistical measure used. However, as we have seen in the CTG example, the value of the agreement coefficient can be very different depending on the weighting scheme or/and the chance definition used. Combining the two last points when interpreting agreement in the CTG example, i.e., ignoring 95% confidence intervals and applying Landis and Koch classification table to all agreement coefficients, the strength of agreement can be qualified as fair using nominal weights, moderate using linear weights and substantial using quadratic weights.

The proportion of disagreement, MAD, MSD and the weighted kappa coefficients provide different and complementary information. Kappa coefficients are scaled measures. They compare the weighted disagreement to the weighted disagreement expected by chance and depend on the distribution of the responses in the sample, leading to what is known as the two kappa paradoxes [26, 40]. On the other hand, the proportion of agreement, MAD and MSD are unscaled measures summarizing the disagreement pattern.

We recommend to report at least one unscaled and one scaled coefficient, both with a confidence interval, because scaled coefficients compare the weighted disagreement to the weighted disagreement expected by chance but do not provide the magnitude of the weighted disagreement itself. It is furthermore advised to report the distribution of the patients in the *K* categories of the scale to explain situations where an unscaled coefficient is large while the corresponding scaled coefficient is low, known as kappa paradox. This property is not longer seen as a paradox but is recognized to be the reflection of the characteristics of the population studied (e.g., [13, 41]). While it is advised to use chance definition 2 in inter-rater agreement studies and chance definition 3 in intra-rater studies, there are no guidelines on the choice of weighting scheme. Nevertheless, in agreement studies, weighted kappa coefficients with linear weights are easier to interpret than with quadratic weights because they are based on the Euclidian distance rather than the squared Euclidian distance. When it concerns reliability, only the quadratic weighted kappa (under chance definitions 2 and 3) can be seen as estimators of reliability. However, reliability is defined based on a linear model (see supplementary file B). While this practice is widely accepted in the literature, defining reliability for an ordinal scale based on a linear model is however questionable because reliability is defined based on variance components. Talking about the variance or the mean of ordinal variables [42] or more generally of bounded outcomes [43] can be inappropriate. Indeed, there is no distance metric defined for ordinal scales. By considering a linear model to define reliability, it is assumed that the measurement level of the scale is at least interval. Moreover, the definition of reliability is based on linear models assuming that the measurement error is symmetrically distributed around the true score of patients and have the same variance whatever the value of the true score [42]. These assumptions are not always reasonable, especially near the boundaries of the scales. Alternative were proposed in the literature but are not presented here because they are not available in common statistical software and seldom used by researchers (see e.g., [44]). Therefore, emphasis was first given to improve the use of common statistical measures. Note that Fleiss kappa corresponds to the weighted kappa coefficient with nominal weights and chance definition 3. It cannot be interpreted as a reliability measure for ordinal scales but well for binary scales [11]. We strongly recommend to report the proportion of patients on the K categories of the scale, the weighting scheme and the chance definition used in order to correctly interpret the agreement/reliability coefficient. Note that other definitions of chance (e.g., [22]), other weighting schemes and other agreement model assumptions (e.g., [45, 46]) have been studied in the literature. They are not presented in this paper because the interpretation of the obtained coefficients is not straightforward.

It is also important to perform sample size calculations to obtain a rough idea of the width of the confidence interval or the power that can be expected given the number of observers and patients in the study. A sample size that is too small can lead to very wide confidence intervals and therefore to an uninformative study. However, it is not possible to have an unique minimal number of patients/objects because of the complexity of the formula for standard errors. In this paper, we propose a method to obtain a rough idea of the magnitude of the minimal number of patients/objects and raters needed. Note that the minimal sample sizes are obtained under the assumption that the distribution of the patients along the ordinal scale is the same for all repeated measurements (over different observers or over the different time points, that is, chance definition 3).

This paper focuses on ordinal scales. In the case of nominal scales, where the categories cannot be ordered, the agreement measures are limited to the proportion of agreement and to the weighted kappa coefficient with nominal weights. Therefore, there is no reliability measure in that case. However, some authors (e.g., [15]) advise dichotomizing the scale by isolating one category from the others to determine the agreement and reliability under that situation. While this may sound appealing, this implies mixing several categories together and assuming that there is no disagreement between them, which cannot be appropriate in all situations.

In summary, we advise researchers to determine the minimum number of observers and patients needed to obtain confidence intervals of reasonable size, report the proportion of patients in all categories of the ordinal scale, report at least one unscaled agreement measure (proportion of (dis)agreement, MAD or MSD) and one scaled agreement measure or a reliability measure (quadratic weighted kappa coefficient), and finally, to report a confidence interval around the agreement and reliability measures. When interpreting quadratic weighted kappa coefficients in terms of reliability, researchers should keep in mind that some mathematical assumptions, not always appropriate, are made.

## Conclusions

The present paper completes the existing GRRAS guidelines by providing more details on various coefficients recommended in agreement and reliability studies and their statistical inference procedures (see Table 2 in [13]). Although limited to statistical measures commonly used by researchers and some extensions thereof, the paper and the shiny application can help researchers improve the methodological quality and reproducibility of studies assessing the reliability and agreement in clinical tests.

## Supplementary Information

Supplementary Material 1. The formula of the weighted coefficients and their standard error are given in the supplementary files A and B. Further assume that for every patient, there is true value, denoted by $T_i$ ($i=1,\cdots ,N$).

## Data Availability

The data, R package and shiny can be downloaded on Github: https://github.com/svanbelle and the Shiny app is directly available following this link: https://svanbelle.shinyapps.io/weightagree/.
