# Supplementary material for: A comprehensive guide to study the agreement and reliability of multi-observer ordinal data
Source: BMC Med Res Methodol. 2024 Dec 20;24:310. doi: 10.1186/s12874-024-02431-y (PMC11660713; doi:10.1186/s12874-024-02431-y)
Supplement: Supplementary file 1 — Supplementary Material 1. The formula of the weighted coefficients and their standard error are given in the supplementary files A and B. Further assume that for every patient, there is true value, denoted by $T_i$Ti ($i=1,\cdots ,N$i=1,⋯,N). [file 12874_2024_2431_MOESM1_ESM.pdf]

A comprehensive guide to study the agreement and reliability of multi-observer ordinal data

Supplementary material

Sophie Vanbelle*[1], Christina Hernandez Engelhart[2,3], and Ellen Blix[3]

[1]Methodology and Statistics, CAPHRI, Maastricht University, P. Debyeplein, 1, Maastricht, 6229 HA, The Netherlands

[2]Norwegian Research Center for Women's Health, Oslo University Hospital, P.O box 4950 Nydalen, Oslo, N-0424, Norway

[3]Faculty of Health Sciences, Oslo Metropolitan University, P.O box 4 St Olavs plass, Oslo, N-0130, Norway

October 24, 2024

*Corresponding author: sophie.vanbelle@maastrichtuniversity.nl

Appendix A Population model for agreement

Let the random variable Y_{ir} denote the classification of patient i ($i = 1, \cdots, N$) by observer r ($r = 1, \cdots, R_i$) on a K-ordinal scale. We assume that the number of observers is the same for all patients ($R_i = R$, $\forall i = 1, \cdots, N$).

Let $P(Y_{is} = j, Y_{it} = k)$ denote the probability for a patient i randomly taken in the patients population $\mathcal{I}$ to be classified in category j by observer s and in category k by observer t ($i = 1, \cdots, N$; $s \neq t = 1, \cdots, R$; $j, k = 1, \cdots, K$). Let first assume that the two observers are randomly taken in a population of observers $\mathcal{R}$.

Agreement and disagreement indexes are defined as the expected value, over the population of patients and the population of observers, of a weighted sum of the probabilities $P(Y_{is} = j, Y_{it} = k)$, i.e., weighted disagreement is defined as

$$Q_{ow} = \sum_{j=1}^{K} \sum_{k=1}^{K} \nu_{jk} E_{\mathcal{IR}} \left(P(Y_{is} = j, Y_{it} = k) \right)$$

where ν_{jk} are disagreement weights. The nominal disagreement weights are defined as $\nu_{jk} = 1 - \delta_{jk}$ where δ_{jk} is the Kronecker delta equal to 1 if $j = k$ or 0 otherwise. Weighted disagreement is then the probability that two randomly taken observers disagree on the classification of a randomly taken patient. Linear disagreement weights are defined as $\nu_{jk} = |k - j|$ and the quadratic disagreement weights are defined as $\nu_{jk} = (k - j)^2$ ($j, k = 1, \cdots, K$). Note that the disagreement weights can be standardized to take values between 0 and 1 by dividing them by the maximum weight, i.e., $\nu_{jk} = |k - j|/(K - 1)$ for the linear weights and $\nu_{jk} = (k - j)^2/(K - 1)^2$ for the quadratic weights.

Using unscaled linear disagreement weights, the weighted disagreement is also called the *mean absolute deviation* ,

$$Q_{ow} = MAD = \sum_{j=1}^{K} \sum_{k=1}^{K} |j - k| E_{\mathcal{IR}} \left(P(Y_{is} = j, Y_{it} = k) \right) = E_{\mathcal{IR}} |Y_{is} - Y_{it}|.$$

The weighted disagreement is the expected Euclidian distance between the ratings of a randomly taken patient by two randomly taken observers. Similarly, using unscaled quadratic disagreement weights, the *mean squared deviation* is obtained,

$$Q_{ow} = MSD = \sum_{j=1}^{K} \sum_{k=1}^{K} (j - k)^2 E_{\mathcal{IR}} \left(P(Y_{is} = j, Y_{it} = k) \right) = E_{\mathcal{IR}} (Y_{is} - Y_{it})^2.$$

The weighted disagreement is the expected squared Euclidian distance between the ratings of a randomly taken patient by two randomly taken observers.

Similarly, the weighted agreement is defined as

$$P_{ow} = \sum_{j=1}^{K}\sum_{k=1}^{K} w_{jk} E_{\mathcal{IR}}\left(P(Y_{is} = j, Y_{it} = k)\right).$$

Agreement weights are usually defined in their scaled version, i.e., $w_{jk} = \delta_{jk}$ for nominal weights, $w_{jk} = 1 - |k-j|/(K-1)$ for linear weights and $w_{jk} = 1 - (k-j)^2/(K-1)^2$ for quadratic weights.

Chance (dis)agreement is determined according to two principles. First, chance (dis)agreement is obtained under the statistical independence assumption of the observers, i.e., $P(Y_{is} = j, Y_{it} = k) = P(Y_{is} = j)P(Y_{it} = k)$. Second, different assumptions are made on the distribution of the scores given by the observers. Common assumptions are

Def 1. The probability for a randomly taken observer to classify a randomly taken patient in the categories of the scale is uniform, i.e., $P(Y_{ir} = j) = 1/K$. That is, it is assumed that patients are classified completely at random on the scale.

Def 2. The probability for a randomly taken patient to be classified in category j of the scale by observer r is equal to the observed proportion of patients classified by observer r in category j, i.e., $P(Y_{ir} = j) = p_{j,r}$.

Def 3. The probability for a randomly taken patient to be classified in category j of the scale by observer r is equal to the overall proportion of patients classified in category j, i.e., $P(Y_{ir} = j) = \frac{1}{R}\sum_{r=1}^{R} p_{j,r}$.

Then, the weighted kappa coefficients are defined as

$$\kappa_{ws} = 1 - \frac{Q_{ow}}{Q_{ews}} = \frac{P_{ow} - P_{ews}}{1 - P_{ews}}.$$

The weighted kappa coefficients take values between -1 and 1. When the observers are fixed, the expected value over the population of observers has to be replaced by the mean over the set of observers.

When two observers classify a sample of N patients or objects on a K-ordinal scale, the observations can be summarized in a $K \times K$ contingency table (see Table 1 in the main manuscript). The estimators

of the population agreement coefficients presented above are obtained by replacing probabilities by the respective sample proportions, as shown below.

The *weighted disagreement* estimator is the weighted sum

$$q_{ow} = \hat{Q}_{ow} = \sum_{j=1}^{K}\sum_{k=1}^{K} v_{jk}p_{jk}, \tag{A1}$$

where v_{jk} are the disagreement weights. In terms of agreement weights, we have

$$p_{ow} = \hat{P}_{ow} = \sum_{j=1}^{K}\sum_{k=1}^{K} w_{jk}p_{jk}. \tag{A2}$$

With the nominal disagreement weights, we obtain respectively the well-known *proportion of disagreement*,

$$q_o = 1 - \sum_{j=1}^{K} p_{jj} \tag{A3}$$

and *proportion of agreement*,

$$p_o = \sum_{j=1}^{K} p_{jj} = 1 - q_o. \tag{A4}$$

Using the unscaled linear disagreement weights, we obtain *the mean absolute deviation* ,

$$\widehat{MAD} = \sum_{j=1}^{K}\sum_{k=1}^{K} |j-k|p_{jk} = \sum_{i=1}^{N} \frac{|y_{i1} - y_{i2}|}{N} \tag{A5}$$

where y_{ir} denotes the category chosen by observer $r = 1, 2$ for patient/object $i = 1, \cdots, N$. When the unscaled quadratic weights are used, *the mean squared deviation* is estimated by

$$\widehat{MSD} = \sum_{j=1}^{K}\sum_{k=1}^{K} (j-k)^2 p_{jk} = \sum_{i=1}^{N} \frac{(y_{i1} - y_{i2})^2}{N}. \tag{A6}$$

The weighted disagreement and agreement expected by chance can be estimated according to definitions 1, 2 or 3 as follows,

$$q_{ew1} = \frac{1}{K^2}\sum_{j=1}^{K}\sum_{k=1}^{K} v_{jk} \text{ and } p_{ew1} = \frac{1}{K^2}\sum_{j=1}^{K}\sum_{k=1}^{K} w_{jk}, \tag{A7}$$

$$q_{ew2} = \sum_{j=1}^{K}\sum_{k=1}^{K} v_{jk}p_{j.}p_{.k} \text{ and } p_{ew2} = \sum_{j=1}^{K}\sum_{k=1}^{K} w_{jk}p_{j.}p_{.k}, \tag{A8}$$

and

$$q_{ew3} = \sum_{j=1}^{K}\sum_{k=1}^{K} v_{jk}\left(\frac{p_{j.}+p_{.k}}{2}\right)^2 \text{ and } p_{ew3} = \sum_{j=1}^{K}\sum_{k=1}^{K} w_{jk}\left(\frac{p_{j.}+p_{.k}}{2}\right)^2. \quad \text{(A9)}$$

The weighted kappa coefficient can then be estimated by

$$\hat{\kappa}_{ws} = 1 - \frac{q_{ow}}{q_{ews}} = \frac{p_{ow}-p_{ew}}{1-p_{ews}} \quad s = 1, 2, 3. \quad \text{(A10)}$$

When there are more than two observers, the weighted (dis)agreement and the weighted (dis)agreement expected by chance are obtained by taking the average over all $R(R-1)$ pairs of observers, i.e.

$$q_{ow} = \frac{1}{NR(R-1)}\sum_{p=1}^{R(R-1)} q_{ow}^{(p)} \text{ and } p_{ow} = \frac{1}{NR(R-1)}\sum_{p=1}^{R(R-1)} p_{ow}^{(p)} \quad \text{(A11)}$$

and

$$q_{ews} = \frac{1}{NR(R-1)}\sum_{p=1}^{R(R-1)} q_{ews}^{(p)}, \text{ and } p_{ews} = \frac{1}{NR(R-1)}\sum_{p=1}^{R(R-1)} p_{ews}^{(p)}, \;\; s = 1, 2, 3 \quad \text{(A12)}$$

where the subscript (p) denotes a pair of observers.

Appendix B Population model for reliability

B.1 Classical test theory

Reliability is a concept with origin in classical test theory [1]. In classical test theory, an observed score Y_i is modeled as the sum of a true score T_i and a measurement error W_i,

$$Y_i = T_i + W_i, \;\; i = 1, \cdots, N$$

where the measurement errors are assumed to have a mean of 0, to be uncorrelated and independent of the true score. The observed scores, true scores and measurement errors are assumed to have variance σ_Y^2, σ_T^2 and σ_W^2, respectively. Reliability is then defined as the squared correlation between the observed score and the true score,

$$cor(Y_i, T_i)^2 = \frac{\sigma_T^2}{\sigma_Y^2} = \frac{\sigma_T^2}{\sigma_T^2 + \sigma_W^2}. \quad \text{(B1)}$$

However, in practice, it is often not possible to know the value of the true score (see e.g., [2] for a thorough discussion). The true score is commonly approached by considering R parallel replicated measurements,

$$Y_{ir} = T_{ir} + W_{ir},\ i = 1, \cdots, N; r = 1, \cdots, R.$$

If, on top on the assumptions of classical test theory, the assumption of parallel measurements is further made, that it, all replicates have the same true score ($E(Y_{ir}) = E(Y_{is}) = T_i$) and equal error variance ($var(W_{ir}) = var(W_{is}) = \sigma_W^2$), then the correlation between two replicated measurements is equal to the reliability, as defined in Eqn. B1,

$$cor(Y_{ir}, Y_{ir'}) = \frac{\sigma_T^2}{\sigma_T^2 + \sigma_W^2}.$$

Depending on how replicated measurements are obtained, reliability takes different names, such as for example, Cronbach α, split-half, test-retest, intra-observer or inter-observer reliability.

B.2 One-way ANOVA model

Under the parallel measurement assumption, data can also be represented in a one-way random effect ANOVA model. Let Y_{ir} denote the score given by observer r ($r = 1, \cdots, R$) on patient i ($i = 1, \cdots, N$). The score can be decomposed as

$$Y_{ir} = \mu + S_i + W_{ir},\ (i = 1, \cdots, N; r = 1, \cdots, R) \tag{B2}$$

where μ is the overall population mean of the measurements, S_i is the deviation of patient i from μ ($\mu + S_i = T_i$) and W_{ir} is a residual component equal to the sum of the non separable effects of the observers, the interaction between the observers and the patients and the error term.

Since $\mu + S_i = T_i$, the variance of S_i is equal to σ_T^2 and the variance of W_{ir} is equal to σ_W^2 ($i = 1, \cdots, N; r = 1, \cdots, R$). Furthermore, S_i are assumed to be independent of W_{ir}. The expected mean squares related to the one-way random effects ANOVA model are given in Table 1.

Table 1: One-way random effects ANOVA model

Variability	Sum of squares	Degrees of freedom	Mean squares	E(MS)
Between patients	BSS	$N-1$	BMS	$R\sigma_T^2 + \sigma_W^2$
Within patients	WSS	$N(R-1)$	WMS	σ_W^2
Total	TSS	$NR-1$		

Under a one-way ANOVA model, reliability corresponds to an intraclass correlation coefficient,

$$\rho = \frac{cov(Y_{ir}, Y_{is})}{\sqrt{var(Y_{ir})var(Y_{is})}} = \frac{\sigma_T^2}{\sigma_T^2 + \sigma_W^2}. \tag{B3}$$

One can see in Table 1 that WMS is an unbiased estimate of σ_W^2 and $(BMS - WMS)/R$ is an unbiased estimate of σ_B^2. The ANOVA estimator of ρ is given by

$$\widehat{\rho} = \frac{BMS - WMS}{BMS + (R-1)WMS}. \tag{B4}$$

This estimator is consistent but biased [3] since the expectation of a ratio is not equal to the ratio of the expectations. Note that while reliability as defined in Eqn. B.3 lies between 0 and 1, it is possible to have estimates that are negative with the ANOVA estimator.

The weighted kappa coefficient with quadratic weights and chance definition 3 can be written [4]

$$\hat{\kappa}_{w3} = \frac{(N-1)BMS/N - WMS}{(N-1)BMS/N + (R-1)WMS}$$

which is a biased estimator of

$$\rho_\kappa = \frac{R\frac{N-1}{N}\sigma_T^2 + \frac{N-1}{N}\sigma_W^2 - \sigma_W^2}{R\frac{N-1}{N}\sigma_T^2 + \frac{N-1}{N}\sigma_W^2 + R\sigma_W^2 - \sigma_W^2}.$$

We can note that ρ_κ is asymptotically equivalent to ρ. This is why $\hat{\kappa}_{w3}$ can be seen as an estimator of ρ for large N.

A representation of the data in a one-way ANOVA model is advised in intra-observer studies or in inter-observer studies where different sets of observers are involved for each patient/object [5] because it is not possible in that case to differentiate between measurement errors and systematic differences between times/observers.

B.3 Two-way ANOVA model

This rather restrictive model was extended under the generalisability theory, to further decompose the error variance according to different factors, named facets, such as observers, times, etc, according to specific characteristics of the study design. One very common extension is when the same set of observers assess a sample of patients. In that case, it is possible to account for possible systematic differences between the observers by considering a two-way random effect ANOVA model

$$Y_{ir} = \mu + S_i + R_r + e_{ir}$$

where R_r is the effect of observer r randomly selected in the population of observers, with variance σ_R^2, e_{ir} represents the error for patient i and rater r with variance σ_E^2 and the other terms were defined in previous section.

The corresponding ANOVA table is given in Table 2.

Table 2: Two-way random effects ANOVA model (MS for mean squares)

Variability	Sum of squares	Degrees of freedom	MS	E(MS)
Between patients	BSS	$N-1$	BMS	$R\sigma_T^2 + \sigma_E^2$
Within patients	WSS			
Between observers	JSS	$(R-1)$	JMS	$N\sigma_R^2 + \sigma_E^2$
Residuals	ESS	$(N-1)(R-1)$	EMS	σ_E^2
Total	TSS	$NR-1$		

Under this two-way ANOVA model, reliability is defined through two different intraclass correlation coefficients, known as the intraclass correlation for consistency and the intraclass correlation for agreement,

$$\rho_C = \frac{\sigma_T^2}{\sigma_T^2 + \sigma_E^2} \quad \text{and} \quad \rho_A = \frac{\sigma_T^2}{\sigma_T^2 + \sigma_R^2 + \sigma_E^2}. \tag{B5}$$

The difference between the two reliability coefficients is in the denominator. In ρ_C, the variance of the observers is ignored. This coefficient is used when researchers are interested in using the measurement instrument only to rank patients/objects because it does not account for the fact that some observers can systematically give lower or higher scores than the others. This possible systematic difference is taken into account in ρ_A, which is more commonly used because we are generally interested in the value given by the measurement instrument rather than only using the measurement instrument to rank patients.

In the above, observers were assumed to be random. When the observers in the study are the only of interest, then observers are modeled through fixed effects. The terms R_r are replaced by a_r under the constraint $\sum_{r=1}^{R} a_r = 0$. The parameter σ_R^2 is replaced by

$$\theta_R^2 = \sum_{r=1}^{R} a_r^2/(R-1). \tag{B6}$$

The estimators of the intraclass correlation coefficients are however the same when observers are considered random or fixed and are given by

$$\widehat{\rho}_A = \frac{BMS - EMS}{BMS + (R-1)EMS + R(JMS - EMS)/N} \tag{B7}$$

and

$$\widehat{\rho}_C = \frac{BMS - EMS}{BMS + (R-1)EMS}. \tag{B8}$$

Here too, while reliability lies between 0 and 1, it is possible to have estimates that are negative with the ANOVA estimator.

When the scale is ordinal, [6] have shown that the quadratic weighted kappa coefficient can be written as

$$\hat{\kappa}_{w2} = \frac{BMS - EMS}{BMS + (R-1)EMS + R\ JMS/(N-1)}$$

and is a biased estimator of

$$\rho_\kappa = \frac{\sigma_T^2}{\sigma_T^2 + \sigma_R^2 + \sigma_e^2 + (\sigma_R^2 + \sigma_e^2)/(N-1)}. \tag{B9}$$

If the number of patients N is large, then the quadratic weighted kappa coefficient estimates

$$\rho_A = \frac{\sigma_T^2}{\sigma_T^2 + \sigma_R^2 + \sigma_e^2} \tag{B10}$$

which is the intraclass correlation coefficient for agreement ρ_A between the ratings of a randomly selected patient by the randomly selected observers. This is why the quadratic weighted kappa coefficient with chance definition 2 is also considered to be a reliability measure.

In classical test theory and in the analysis of variance, there is no need for assumptions about the shape of the distribution of the effects S_i, R_j or the errors to define reliability. Such assumptions (e.g., a normal distribution) are nevertheless needed to make statistical inference (e.g., based on the F-distribution [5]).

Appendix C Large sample variance using the delta method

The large sample variance of the various agreement coefficients defined in Appendix A were derived using the delta method. The formulas for MAD and MSD, are, to the best of our knowledge, not yet presented in the literature and are generalisations of the formula for the proportion of agreement

derived in [7].

C.1 Two-way ANOVA model

Under the two-way ANOVA model, we assume that the same R observers classify a sample of N patients or objects on a K-ordinal scale. The large sample variance of the proportion of agreement, MAD and MSD can be estimated by using

$$\widehat{var(p_{ow})} = \frac{1}{N^2}(\sum_{i=1}^{N}\sum_{r=1}^{R}\sum_{t\neq r} w_{i_r,i_t}^2 - Np_{ow}^2) \tag{C1}$$

and

$$\widehat{var(q_{ow})} = \frac{1}{N^2}(\sum_{i=1}^{N}\sum_{r=1}^{R}\sum_{t\neq r} \nu_{i_r,i_t}^2 - Nq_{ow}^2) \tag{C2}$$

where the weights ν_{i_r,i_t} (resp. w_{i_r,i_t}) are disagreement (resp. agreement) weights corresponding to the cell where patient/object i was classified by observer r and observer t.

[8] and [9] derived the large sample variance of the weighted kappa coefficients using the delta method. This gives for the weighted kappa coefficients under the different chance definitions $s = 1, 2, 3$,

$$var(\hat{\kappa}_{ws}) = \frac{\sum_{i=1}^{N}((1-p_{ews})p_{ow,i} - 2(1-p_{ow})p_{ews,i})^2/N - (p_{ow}p_{ews} - 2p_{ews} + p_{ow})^2}{N(1-p_{ews})^4} \tag{C3}$$

where

$$p_{ow,i} = \sum_{r=1}^{R}\sum_{t\neq r} w_{i_r,i_t}/(R(R-1)). \tag{C4}$$

Under chance definition 2, we have

$$p_{ews,i} = \sum_{r=1}^{R}\sum_{t\neq r} w_r(i_t)/(R(R-1)) \tag{C5}$$

where

$$w_r(i_t) = \sum_{k=1}^{K} w_{k,i_t} p_{k,r}$$

and where $p_{k,r}$ is the proportion of patients assigned to category k by observer r and w_{k,i_t} the agreement weight corresponding to category k and the category where observer t classified patient i.

C.2 One-way ANOVA

Let m_{ij} denote the number of observers who classified patient or object i in category j ($i = 1, \cdots, N$; $j = 1, \cdots, K$). By defining the following quantities

$$P_{jk} = \frac{1}{NR(R-1)} \sum_{i=1}^{N} m_{ij} m_{ik} \text{ when } j \neq k \ (j, k = 1, \cdots, K) \tag{C6}$$

and

$$P_{jj} = \frac{1}{NR(R-1)} \sum_{i=1}^{N} m_{ij}(m_{ij} - 1) \text{ for } j = 1, \cdots, K, \tag{C7}$$

the observed weighted disagreement and agreement are respectively equal to

$$q_{ow} = \sum_{j=1}^{K} \sum_{k=1}^{K} v_{jk} P_{jk} \text{ and } p_{ow} = \sum_{j=1}^{K} \sum_{k=1}^{K} w_{jk} P_{jk} \tag{C8}$$

and

$$q_{ew3} = \sum_{j=1}^{K} \sum_{k=1}^{K} v_{jk} P_j P_k \text{ and } p_{ew3} = \sum_{j=1}^{K} \sum_{k=1}^{K} w_{jk} P_j P_k \tag{C9}$$

where P_j is the overall proportion of patients or objects in category j ($j = 1, \cdots, K$).

The large sample variance is then computed by replacing respectively p_{jk} by P_{jk} and $p_{j.}, p_{.k}$ by P_j and P_k in Equations C3 and C1. Furthermore, we need

$$p_{ow,i} = \sum_{j=1}^{K} \sum_{k \neq j}^{K} w_{jk} m_{ij} m_{ik} / (R(R-1)) + \sum_{j=1}^{K} w_{jj} m_{ij}(m_{ij} - 1)/(R(R-1))$$

and

$$p_{ew3,i} = \sum_{j=1}^{K} \sum_{k=1}^{K} w_{jk} m_{ij} P_k / R.$$

Note that the value of the variances obtained will be the same as the one obtained by the general formula under chance definition 3.

C.3 Fisher-Z transform

Using Fisher-Z transform is known to improve the statistical properties (e.g. the coverage) of confidence intervals for kappa coefficients (see e.g., [10, 11]). The idea is to derive a confidence interval for

$$\frac{1}{2} ln \left(\frac{1 + \hat{\kappa}_{ws}}{1 - \hat{\kappa}_{ws}} \right) = artanh(\hat{\kappa}_{ws}), \ (-1 < \hat{\kappa}_{ws} < 1).$$

and then transform it back to the original scale. The lower and upper bounds of the $(1 - \alpha)100\%$

confidence interval are given by

$$L, U = \frac{1}{2} ln \left(\frac{1 + \hat{\kappa}_{ws}}{1 - \hat{\kappa}_{ws}} \right) \pm Q_Z(1 - \alpha/2) \frac{1}{\sqrt{1 - \hat{\kappa}_{ws}^2}} \sqrt{var(\hat{\kappa}_{ws})} \; (-1 < \hat{\kappa}_{ws} < 1)$$

where $Q_z(1-\alpha/2)$ is the $(1-\alpha)\times$ 100% percentile of the standard normal distribution, and $\hat{\kappa}_{ws}$ and $var(\hat{\kappa}_{ws})$ were previously defined. Then, the inverse of Fisher Z-transform of the lower (L) and the upper (U) bounds is taken to form the $(1-\alpha)100\%$ confidence interval for the weighted kappa coefficient,

$$\frac{exp(2L) - 1}{exp(2L) + 1}; \frac{exp(2U) - 1}{exp(2U) + 1}.$$

References

[1] Spearman C. The Proof and Measurement of Association between Two Things. The American Journal of Psychology. 1904;15(1):72-101. Available from: http://www.jstor.org/stable/1412159.

[2] Vach W, Gerke O. How Replicates Can Inform Potential Users of a Measurement Procedure about Measurement Error: Basic Concepts and Methods. Diagnostics. 2021;11(2). Available from: https://www.mdpi.com/2075-4418/11/2/162.

[3] Olkin M, Pratt JW. Unbiased estimation of certain correlation coefficients. Annals of Mathematical Statistics. 1958;29:201-11.

[4] Janson H, Olsson U. A Measure of Agreement for Interval or Nominal Multivariate Observations by Different Sets of Judges. Educational and Psychological Measurement - EDUC PSYCHOL MEAS. 2004 02;64:62-70.

[5] McGraw KO, Wong SP. Forming inferences about some intraclass correlation coefficients. Psychological Methods. 1996;1(1):30-46.

[6] Fleiss JL, Cohen J. The equivalence of weighted kappa and the intraclass correlation coefficient as measure of reliability. Educational and Psychological Measurement. 1973;33:613-9.

[7] Vanbelle S. Statistical inference for agreement between multiple raters on a binary scale. British Journal of Mathematical and Statistical Psychology. 2024;77(2):245-60. Available from: https://bpspsychub.onlinelibrary.wiley.com/doi/abs/10.1111/bmsp.12333.

[8] Schouten HJA. Measuring pairwise agreement among many observers. II. Some improvements and additions. Biometrical Journal. 1982;24:497-504.

[9] O'Connell DL, Dobson AJ. General observer-agreement measures on individual subjects and groups of subjects. Biometrics. 1984:973-83.

[10] Kraemer HC. Ramifications of a population model for κ as a coefficient of reliability. Psychometrika. 1979;44:461-72.

[11] Moss J. Measures of Agreement with Multiple Raters: Fréchet Variances and Inference. Psychometrika. 2024;89(2):517-41.
